# *Ct*GEM typing: Discrimination of *Chlamydia trachomatis* ocular and urogenital strains and major evolutionary lineages by high resolution melting analysis of two amplified DNA fragments

**DOI:** 10.1371/journal.pone.0195454

**Published:** 2018-04-10

**Authors:** Philip M. Giffard, Patiyan Andersson, Judith Wilson, Cameron Buckley, Rachael Lilliebridge, Tegan M. Harris, Mariana Kleinecke, Kerry-Ann F. O’Grady, Wilhelmina M. Huston, Stephen B. Lambert, David M. Whiley, Deborah C. Holt

**Affiliations:** 1 Global and Tropical Health Division, Menzies School of Health Research, Charles Darwin University, Darwin, Northern Territory, Australia; 2 School of Psychological and Clinical Sciences, Charles Darwin University, Darwin, Northern Territory, Australia; 3 Faculty of Medicine, Centre for Clinical Research, The University of Queensland, Herston, Queensland, Australia; 4 Child Health Division, Menzies School of Health Research, Charles Darwin University, Darwin, Northern Territory, Australia; 5 Centre for Children’s Health Research, Institute of Health and Biomedical Innovation, Queensland University of Technology, Brisbane, Queensland, Australia; 6 School of Life Sciences, University of Technology, Sydney, New South Wales, Australia; 7 UQ Child Health Research Centre, The University of Queensland, Brisbane, Queensland, Australia; 8 Pathology Queensland Central Laboratory, Brisbane, Queensland, Australia; Universita degli Studi di Bologna, ITALY

## Abstract

*Chlamydia trachomatis* infects the urogenital tract (UGT) and eyes. Anatomical tropism is correlated with variation in the major outer membrane protein encoded by *ompA*. Strains possessing the ocular *ompA* variants A, B, Ba and C are typically found within the phylogenetically coherent “classical ocular lineage”. However, variants B, Ba and C have also been found within three distinct strains in Australia, all associated with ocular disease in children and outside the classical ocular lineage. *Ct*GEM genotyping is a method for detecting and discriminating ocular strains and also the major phylogenetic lineages. The rationale was facilitation of surveillance to inform responses to *C*. *trachomatis* detection in UGT specimens from young children. *Ct*GEM typing is based on high resolution melting analysis (HRMA) of two PCR amplified fragments with high combinatorial resolving power, as defined by computerised comparison of 65 whole genomes. One fragment is from the hypothetical gene defined by Jali-1891 in the *C*. *trachomatis* B_Jali20 genome, while the other is from *ompA*. Twenty combinatorial *Ct*GEM types have been shown to exist, and these encompass unique genotypes for all known ocular strains, and also delineate the TI and T2 major phylogenetic lineages, identify LGV strains and provide additional resolution beyond this. *Ct*GEM typing and Sanger sequencing were compared with 42 *C*. *trachomatis* positive clinical specimens, and there were no disjunctions. *Ct*GEM typing is a highly efficient method designed and tested using large scale comparative genomics. It divides *C*. *trachomatis* into clinically and biologically meaningful groups, and may have broad application in surveillance.

## Introduction

Whole genome-based studies have shown that *Chlamydia trachomatis* encompasses four major evolutionary lineages [1–4]. The earliest divergence corresponds to strains associated the invasive lymphogranuloma venereum (LGV) sexually transmitted infections (STI). The remainder of the species is divided into the T1 and T2 lineages. A coherent lineage within T2 is composed of trachoma-associated strains from Africa and East Asia, and is termed here the “classical ocular lineage”. Remaining T2 strains and all T1 strains are associated with non-invasive STIs.

It has long been common practice to define sub-groups within the species *C*. *trachomatis* in accordance with variation in *ompA* [5–8] which encodes the major outer membrane protein. *OmpA* confers variation in immunoreactivity, is associated with tropism towards the urogenital tract (UGT) or ocular sites, and pathogenic properties. Specifically, the “LGV” *ompA* genotypes L1, L2, and L3 are LGV associated, the UGT genotypes D, E, F, G, H, Ia, J, Ja and K are associated with the non-invasive *C*. *trachomatis* STIs, and the “ocular” genotypes A, B, Ba and C are associated with trachoma and similar ocular disease exhibiting characteristic “face to face” transmission amongst children. There is significant *ompA* homoplasy with respect to evolutionary lineages defined by genome wide SNPs [1–4, 9]. Of particular relevance to this study, the ocular *ompA* genotypes B, Ba and C are not only found in the classical ocular lineage, but also in other lineages isolates from northern Australia, from children with ocular disease, and strongly associated with the ocular site [2]. These Australian strains are not within the classical ocular lineage. Three strains were identified. Two were *ompA* genotypes Ba and C (termed AusBa and AusC). On the basis of genome wide orthologous SNPs, these are closely related to each other within the T1 lineage and also closely related to UGT strains of *ompA* genotypes E and F. The third Australian strain is *ompA* genotype B (termed AusB). AusB is unrelated to AusBa and AusC and is within the T2 lineage, closely related to UGT strains of *ompA* genotypes G, H, K and D. A very recent large-scale study of global *C*. *trachomatis* genome diversity has shown that while the AusBa and AusC clones have as yet been found only in Australia, isolates essentially identical to AusB have been found in Finland and The Netherlands [3].

The objective of this study was to develop an efficient *C*. *trachomatis* genotyping method that i) discriminated ocular lineages from UGT lineages, ii) discriminated the classical ocular lineage and the three Australian ocular lineages from each other, and iii) discriminated the major evolutionary lineages of the entire species. The proximate rationale was the enhancement of the evidence base for interpreting instances of *C*. *trachomatis* detection in young children in remote northern Australia. The issue of child sexual abuse in remote northern Australia has a high public profile and is socio-politically contentious [10]. Reports of abuse led to high profile Australian government interventions [11]. However, the extent and nature of these behaviours are poorly understood, and the potential for Aboriginal individuals, families and communities to be unjustly stereotyped is obvious [10]. While the detection of an STI agent in a UGT specimen from a young child is normally regarded as highly suggestive of abuse [12–14], it is difficult to rule out other mechanisms. In particular, the notion of autoinoculation or contamination of the UGT site from an ocular infection is plausible, particularly in areas where trachoma remains endemic. Our conceptual framework is that on-going genotype surveillance of *C*. *trachomatis* in UGT specimens in the adult/post pubescent population will provide a comparator for genotypes found in young children, when instances of possible abuse are being investigated [15, 16]. Of particular significance is that a 1980s-90s survey in the remote Northern Territory of Australia, revealed that ocular serovar B isolates were frequently found in adult cervical swabs, strongly suggesting transmission of this serovar through sexual networks [2, 17, 18]. In a much more recent survey that encompassed the same part of Australia [16], ocular strains were not detected in adult UGT specimens, indicating that serovar B strains were no longer being transmitted in sexual networks at a detectable frequency. We reason that if ocular strains are absent from adult sexual networks, then an ocular genotype *C*. *trachomatis* from a paediatric UGT specimen does not provide evidence for contact with adult sexual networks and may be suggestive of autoinoculation, or of similar processes that drive trachoma transmission. Conversely, if nominally ocular strains are prevalent in sexual networks, then the genotype of the strain in a paediatric specimen is less informative of origin [15].

The technology platform chosen was high resolution melting analysis (HRMA) [19–21], because of its simplicity and low cost, and the generic instrumentation. The genotyping method we developed meets the performance requirements specifications and is suitable for the surveillance task envisaged. Its simplicity, informative power and low cost suggests that it has wider applicability.

## Results

### Method development

**Fig 1:**
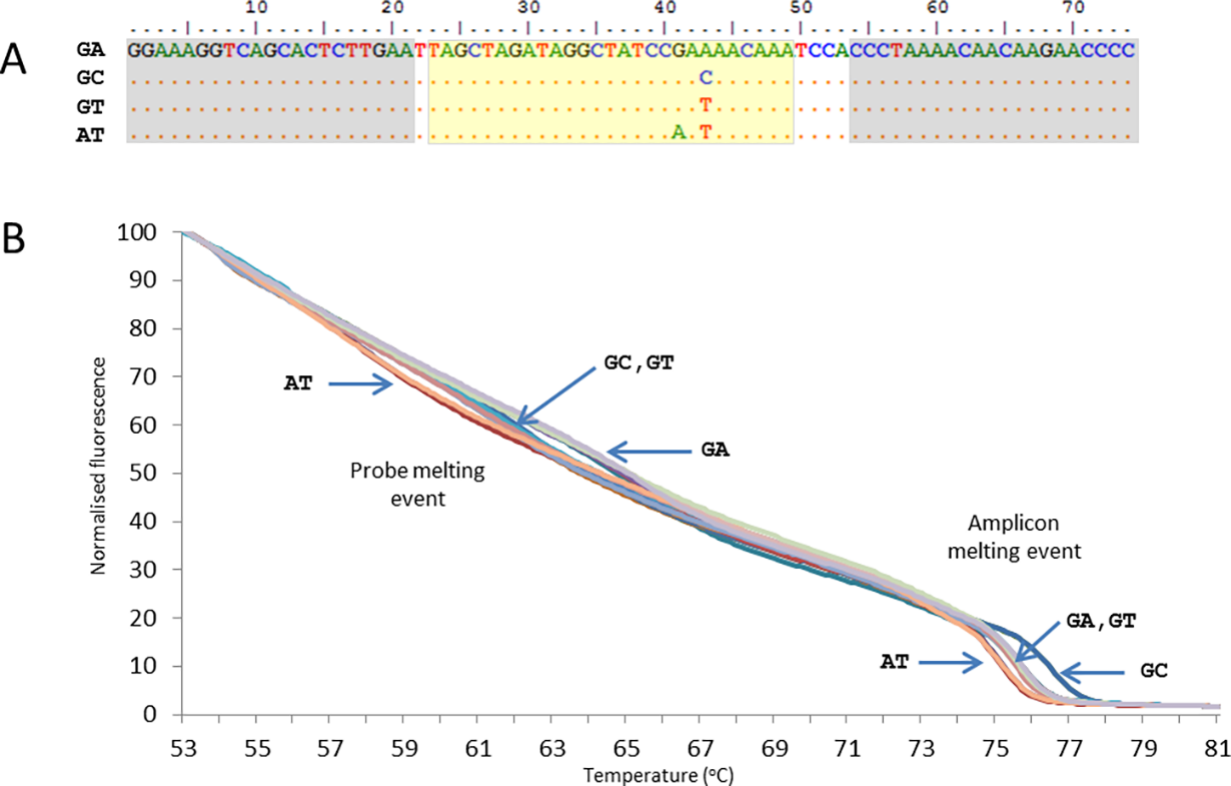
HRMA analysis of the region 1 fragment (rg1). (A) Sequence alignment of the four known haplotypes of rg1 showing the primer sequences (grey shading) and probe sequence (yellow shading). (B) HRMA curves showing the resolution of the four haplotypes.

The conceptual and technical basis of the method resembles “Minim typing’ which makes use of HRMA of fragments within multilocus sequence typing (MLST) loci that are nucleated by SNPs selected for high combinatorial resolving power [22–25]. However, in this study we derived SNPs from whole genome information rather than MLST data. A detailed account of the development of the method is provided (S1 File) In brief, an alignment of alleles at genome-wide orthologous SNPs derived from 65 representative *C*. *trachomatis* genomes was mined for SNP combinations with high Simpsons Index of Diversity using the software Minimum SNPs [26, 27]. It was found that maximal resolving power from two SNPs was obtained when one of the SNPs was the tri-allelic SNP defined by position 222834 in the *C*. *trachomatis* B_Jali20 genome [28] (NC_012686.1), and the other was any of a number of SNPs within *ompA*. This led us to conjecture that a method encompassing both these loci would identify ocular strains and discriminate major phylogenetic groups. The SNP at position 222834 is within a hypothetical gene (Jali-1891) that is annotated as encoding a hypothetical protein that is likely membrane located [28]. There is another SNP at position 222832, and together these define four haplotypes of GA, GT, GC and AT (shown in order of 222832-G/A, 222834-A/T/C). Robust HRMA-based discrimination of these haplotypes was achieved, with the adoption of a nested PCR format in which the second round PCR was an asymmetric PCR which incorporated an unlabelled probe (Fig 1). We term the amplified region “region 1’ which is abbreviated to “rg1”.

**Fig 2:**
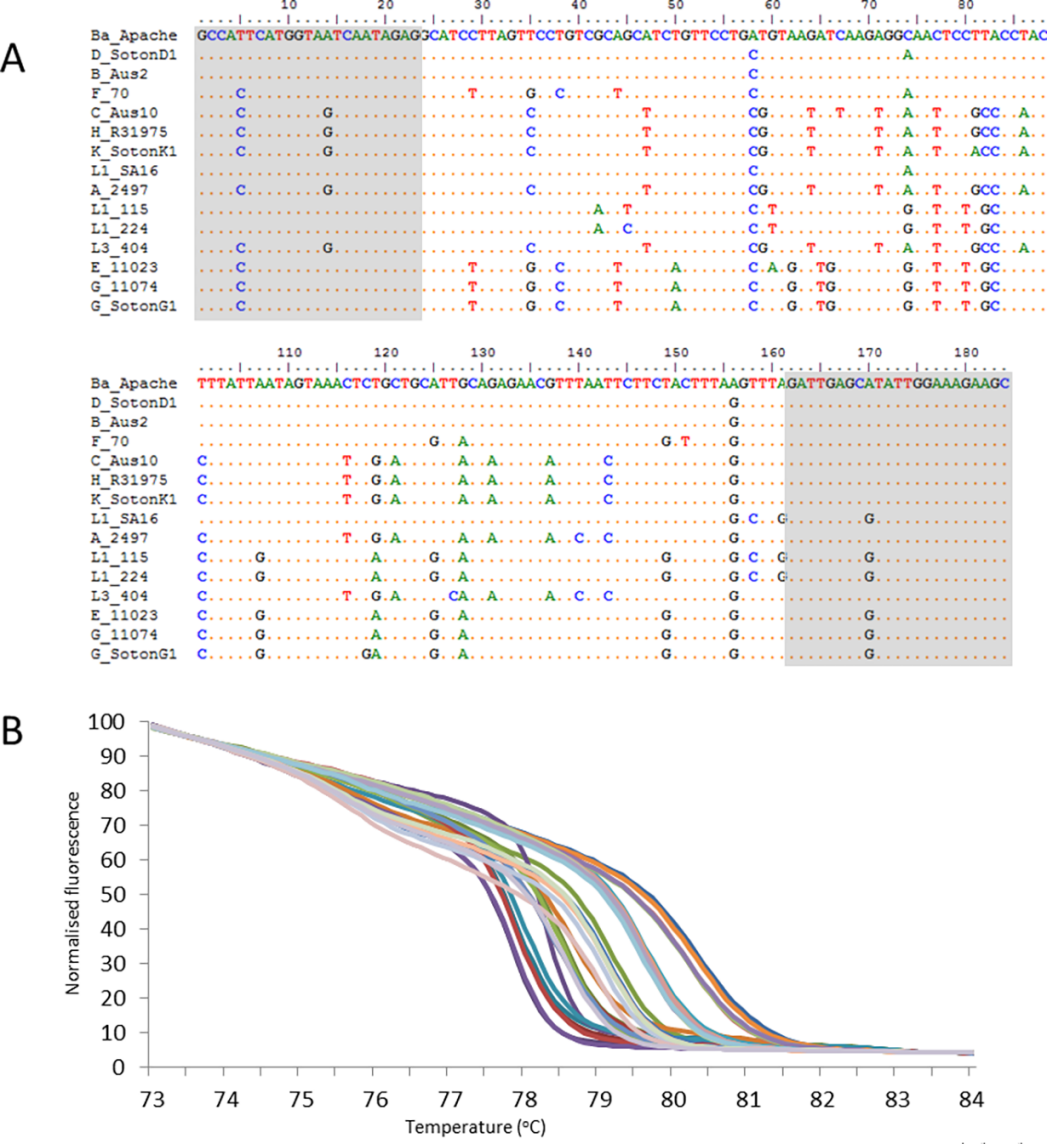
HRMA analysis of the *ompA* fragment (ofr). (A) Sequence alignment of the known haplotypes of ofr showing the primer sequences (grey shading). (B) HRMA curves showing the resolution of the genotypes. A detailed guide for the HRMA is given in S2 File in the supplemental material.

To develop an HRMA based on *ompA*, we determined that a fragment used in a previously described HRMA assay to discriminate *ompA* genotypes B and Ba [16] may have broader applicability for discriminating *ompA* variants. This fragment encompasses the ompA region that encodes the OmpA variable domain 3. A semi-nested PCR and HRMA assay was developed using control strains that divided the *ompA* genotypes into the following groups: Ba+D, B+F, C+H+Ia+J+Ja+K, L1+L2, A, E and G (Fig 2). At this time L3 variants remain untested, but are predicted to produce a unique HRM curveWe termed the amplified fragment “*ompA* fragment” which is abbreviated to “ofr”. An alignment of variation in the ofr is shown in Fig 2. A detailed procedure for assigning curves to HRMA alleles is shown in S2 File.

We elected to number the rg1-ofr combinatorial genotypes from 1–32, in accordance with all possible combinations of the four HRMA alleles of rg1 and eight HRMA alleles of the ofr, even though all 32 genotypes may not exist in nature. We named the procedure “*C**hlamydia* *t**rachomatis* genotyping by melting” *(Ct*GEM), and refer to the genotypes from this method as *Ct*GEM types 1–32. Detailed information regarding the *Ct*GEM types, and their relationship to the phylogeny and *ompA* allelic status are provided in Table 1.

**Table 1:** *Ct*GEM types defined by 559 genomes.

	HRMA alleles of ofr							
Rg1 haplotype	BaD	BF	CHIJJaK	Lx	A	L3	E	G
**GA**	**Type 1, T2** “D in T2”	**Type 2**, **T2** “Ocular AusB”	**Type 3**, **T2** “Non-ocular T2, not B, D or G”	**Type 4,** Not found	**Type 5,** Not found	**Type 6,** Not found	**Type 7**, **T1,** “GA E”	**Type 8**, **T2,** “Typical G”
**GT**	**Type 9, T1** “Ocular AusBa”	**Type 10, T1** “GT F”	**Type 11**, **T1** “Ocular AusC”	**Type 12,** Not found	**Type 13,** Not found	**Type 14,** Not found	**Type 15**, **T1,** “Typical E”	**Type 16,** Not found
**AT**	**Type 17, T1,** “D in T1”	**Type 18, T1,** “AT F”	**Type 19**, **T1,** “T1 J”	**Type 20,** Not found	**Type 21,** Not found	**Type 22,** Not found	**Type 23**, **T1,** “AT E”	**Type 24**, **T1,** “AT G”
**GC**	**Type 25, T2,** “Ocular classical Ba”	**Type 26, T2,** “Ocular classical B”	**Type 27**, **T2,** “Ocular classical C”	**Type 28, LGV,** “LGV L1, L2, L2a, L2b”	**Type 29**, **T2,** “Ocular Classical A”	**Type 30, LGV,** “LGV L3”	**Type 31,** Not found	**Type 32,** Not found

### *In silico* assessment of assay performance

**Fig 3:**
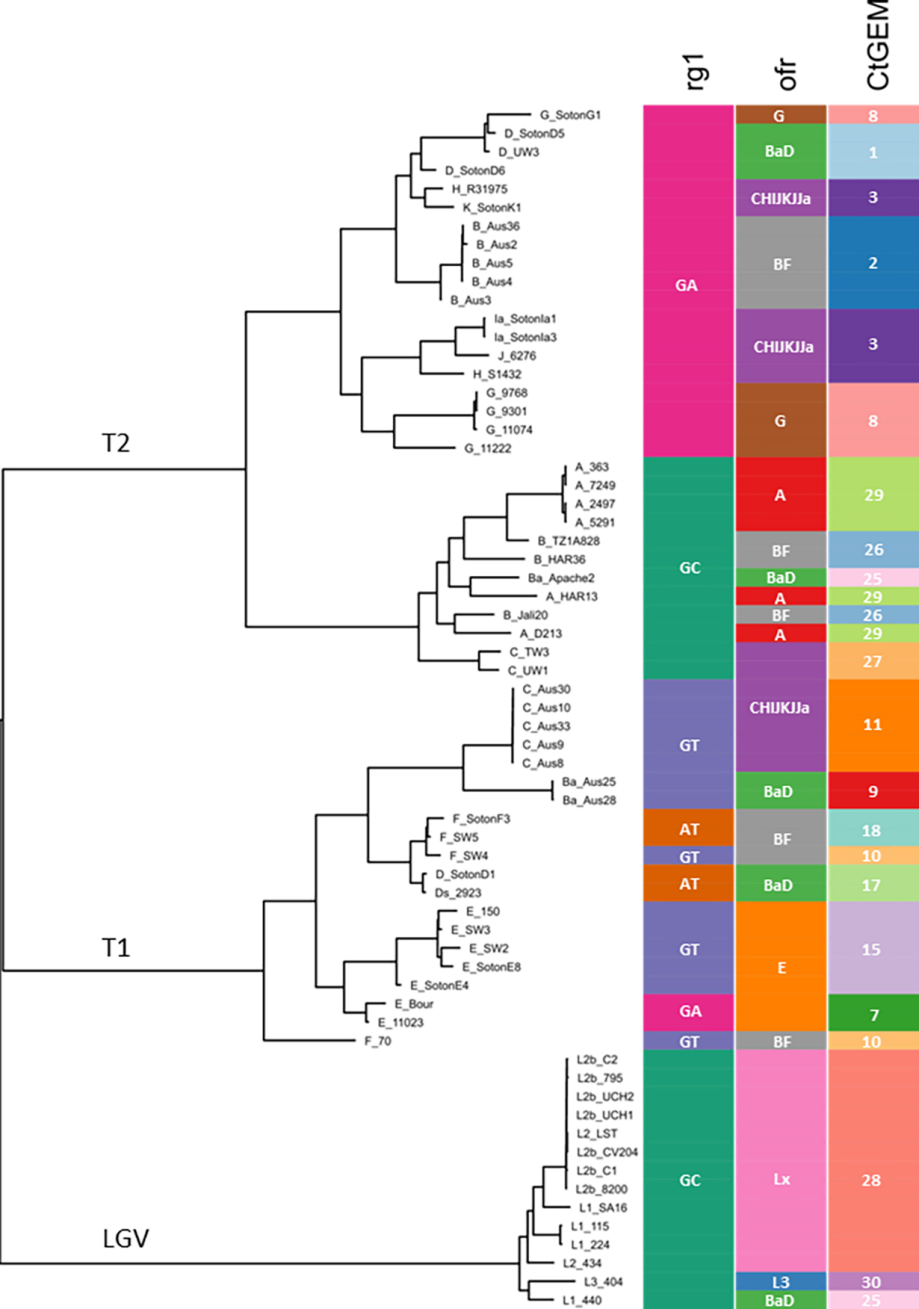
Relationship between rg1 and HRMA alleles alone and in combination, and *C*. *trachomatis* phylogeny. The phylogenetic tree is based on genome-wide orthologous SNPs and adapted from Andersson et al (2). HRMA alleles and *Ct*GEM types are indicated with colours.

The availability of many whole genome data sets enabled us to assess *in silico* the performance of *Ct*GEM typing across known diversity of the species. First, we determined how the genotyping assay would be predicted to resolve the 65 genomes used to design the *Ct*GEM typing. It can be seen in Fig 3 that that the predicted performance of the assay meets requirements. The 20 *Ct*GEM types defined by these genomes encompass four *Ct*GEM types containing only “classical ocular” genomes, and three *Ct*GEM types containing only Australian ocular B, Australian ocular Ba, and Australian ocular C genomes. In intuitive terms, rg1 resolves *C*. *trachomatis* into the T2 lineage (haplotype GA), the T1 lineage (haplotype GT, AT and rare occurrences of GA), and the classical ocular lineage plus the L1 and L2 lineages (haplotype GC). It is fortuitous that the combinatorial resolving power of the two fragments is what is required for the method to meet the performance requirements. In particular, the Australian ocular genomes are resolved from their close UGT-associated relatives on the basis of *ompA* HRMA types. Also fortuitous are the distinctive *ompA* HRMA curves we obtained from *ompA* genotypes E, A and L1/L2. Upon careful analysis of the sequence data we noted that the LGV associated L1-440 has an aberrant ofr sequence that is predicted to yield an HRMA curve indistinguishable from the ofr Ba+D curve. Because L1-440 possesses the rg1 GC haplotype, this strain is predicted to be *Ct*GEM type 25, as for *ompA* genotype Ba strains in the classical ocular lineage, and accordingly should be regarded a false positive for an ocular strain.

For the second stage of the *in silico* experiments, we extended the analyses to a much larger recently published set of whole genome information of *C*. *trachomatis* isolates, comprising the 65 genomes we had already considered and 494 additional genomes (Table 2). Two questions were addressed. First we looked for additional *in silico* inferred *Ct*GEM types that consist of previously unseen combinations of known sequence variants i.e. *Ct*GEM types encompassed by the *Ct*GEM nomenclature but not known to exist prior to this analysis. Second, we searched for previously unidentified SNPs that have potential to confound the HRMA’s we have developed. The rg1 and ofr sequence was determined from the available short read sequencing data using ARIBA [29]. The isolates overwhelmingly conformed to relationship between *Ct*GEM type, phylogenetic position, and tropism previously defined by the 65 genomes. However novel recombinants were identified. Eleven of the strains with non-ocular *ompA* genotypes would be predicted to generate a false positive in a screen for classical or Australian ocular isolates. *OmpA* genotype F strains 11–96 and Fin106 possess the rg1 GA haplotype (*Ct*GEM type 2), as does AusB. *OmpA* genotype D isolates NL4, NL5, NL6, NL8, NL19, Fin163 and S2761 have the rg1 haplotype GT (*Ct*GEM type 9), as does AusBa. *OmpA* genotype J strains J_NL55 and J_Sou106 possess the rg1GT haplotype (*Ct*GEM type 11), as does AusC. No novel SNPs were identified in region 1. Novel SNPs in the HRMA fragments were extremely rare and in only one instance was predicted on the basis of G+C content to impact the HRMA curve. This was strain S4641, which possesses a novel *ompA* sequence for which it is unclear whether to classify as genotype E or genotype G. The ofr sequence is predicted to yield a curve indistinguishable from *ompA* genotype G. The rg1 haplotype is AT, so the predicted *Ct*GEM result is type 24. Strain S4641 is the only known or putative representative of *Ct*GEM type 24.

**Table 2:** Descriptions of the *Ct*GEM types, and the numbers and frequencies of the types in sets of genomes/clinical specimens.

*Ct*GEM type	HRMA alleles	Description	Clade	Global set of genomes ^a^ N (%)	Clinical specimens, Queensland N (%)
1	rg1GA-ofrBaD	D in T2	T2	39 (7.0)	8 (13.8)
2	rg1GA-ofrBF	Ocular AusB	T2	11^a^ (2.0)	0
3	rg1GA- ofrCHIaJJaK	Non-ocular T2 not D or G	T2	88 (15.7)	12 (20.7)
4	rg1GA-ofrLx	Not found	-	0	0
5	rg1GA-ofrA	Not found	-	0	0
6	rg1GA-ofrL3	Not found	-	0	0
7	rg1GA-ofrE	GA E	T1	32 (5.7)	2 (3.5)
8	rg1GA-ofrG	Typical G	T2	59 (10.6)	4 (6.9)
9	rg1GT-ofrBaD	Ocular AusBa	T1	9^b^ (1.6)	1^c^ (1.7)
10	rg1GT-ofrBF	GT F	T1	29 (5.2)	3 (5.2)
11	rg1GT- ofrCHIaJJaK	Ocular AusC	T1	7^d^ (1.3)	1^e^ (1.7)
12	rg1GT-ofrLx	Not found	-	0	0
13	rg1GT-ofrA	Not found	-	0	0
14	rg1GT-ofrL3	Not found	-	0	0
15	rg1GT-ofrE	Typical E	T1	105 (18.8)	16 (27.6)
16	rg1GT-ofrG	Not Found	-	0	0
17	rg1AT-ofrBaD	D in T1	T1	13 (2.3)	1 (1.7)
18	rg1AT-ofrBF	AT F	T1	37 (6.6)	8 (13.8)
19	rg1AT- ofrCHIaJJaK	T1 J	T1	1 (0.2)	1 (1.7)
20	rg1AT-ofrLx	Not found	-	0	0
21	rg1AT-ofrA	Not found	-	0	0
22	rg1AT-ofrL3	Not found	-	0	0
23	rg1AT-ofrE	AT E	T1	9 (1.6)	1 (1.7)
24	rg1AT-ofrG	AT G	T2	1 (0.2)	0
25	rg1GC-ofrBaD	Ocular classic Ba	Ocular	2^f^ (0.4)	0
26	rg1GC-ofrBF	Ocular classic B	Ocular	5 (0.9)	0
27	rg1GC- ofrCHIaJJaK	Ocular classic C	Ocular	3 (0.5)	0
28	rg1GC-ofrLx	LGV L1,L2,L2a	LGV	56 (10.0)	0
29	rg1GC-ofrA	Ocular classic A	Ocular	52 (9.3)	0
30	rg1GC-ofrL3	LGV L3	LGV	1 (0.2)	0
31	rg1GC-ofrE	Not found	-	0	0
32	rg1GC-ofrG	Not found	-	0	0
**Total**				**559**	**58**

^a^ The 11 *Ct*GEM type 2 genomes in the global set encompass nine ocular AusB isolates and two false positives for this strain.

^b^ The nine *Ct*GEM type 9 genomes in the global set encompass two ocular AusBa isolates and seven false positives for this strain.

^c^ The single *Ct*GEM type 9 strain in the clinical specimens is a false positive for the AusBa strain.

^d^ The seven *Ct*GEM type 11 genomes in the global set encompass five ocular AusC isolates and two false positives for this strain.

^e^ The single *Ct*GEM type 11 strain in the clinical specimens is a false positive for the AusC strain.

^f^ The two *Ct*GEM type 25 genomes in the global set encompasses one classical ocular Ba isolate and one false positive for this strain.

The number of predicted *Ct*GEM false positives for ocular strains is 12; one from the 65 genome set and a further 11 from the additional 494 genomes, as indicated above. Of the 559 genomes in the total set, 89 have ocular *ompA* genotypes, so there are 470 true negatives for ocular strains. Specificity = true negatives/(true negatives + false positives), so this equates to a specificity of 0.975 if *Ct*GEM typing were used to screen the 559 genomes for ocular strains. Six of the false positives are “NL” strains from the Netherlands. The equivalent specificity for “NL’ designated isolates alone is 0.902, showing that there is potential for regional variation.

The inferred sensitivity for ocular strains is 1.0, primarily because of absolute linkage between the classical ocular and L lineages, and the rg1 GC haplotype.

It was concluded that *Ct*GEM typing enables screening for ocular strains with high sensitivity and specificity, and also provides a low resolution but robust indication of evolutionary position as defined by genome wide orthologous SNP-based phylogenetic inference.

### Testing using clinical specimens

The robustness of *Ct*GEM typing was tested using 113 *C*. *trachomatis* positive UGT clinical specimens, obtained from public pathology service providers in the Australian state of Queensland. To generate a gold standard comparator, the regions subjected to HRMA were first analysed by Sanger sequencing of PCR fragments, in a different laboratory to where the *Ct*GEM typing was performed. For both targets, specimens were tested either once or twice. For rg1, a subset of specimens where genotyping failed was re-tested, but this did not increase the success rate. For ofr, a subset of specimens that initially failed to be genotyped was re-tested using a reaction that incorporated a small reduction in the primer annealing temperature (the conditions for the repeat reactions are the conditions incorporated in the final definitive procedure). This resulted in six initially “fail” specimens being successfully genotyped.

The results are summarised in Table 3, and complete results are in S3–S5 Files. There were no inconsistencies between HRMA and Sanger sequencing. Also, the *Ct*GEM types obtained and their relative abundances were similar to that shown by the *in silico* analysis of genome sequences. The predominant mode of failure was complete amplification failure, and there was considerable consistency between failure of HRMA and failure of sequence determination (Table 3), suggesting that failure was due to low target concentration or PCR inhibition. For three specimens (58, 77 and 83), complete failure of rg1 amplification was not observed, but probe melting was not sufficiently clear to reliably discriminate between the GA and GT haplotypes. It was of interest that only two of the specimens yielded *Ct*GEM types consistent with ocular strains. Specimen 81 is *Ct*GEM type 9, which corresponds to the AusBa strain. The initial attempt to obtain sequence from specimen 81 was unsuccessful. However, we made additional efforts to sequence the HRMA loci and it was determined that it was *ompA* genotype D and so a false positive for an ocular strain. Specimen 96 yielded *Ct*GEM type 11 which corresponds to the AusC strain. However, the *ompA* sequence indicated that it was *ompA* genotype Ja, and so also a false positive for an ocular strain. Complete *Ct*GEM types were obtained by *Ct*GEM typing and/or sequencing from 58 clinical specimens and none have ocular *ompA* genotypes.

**Table 3:** Numbers and percentages of successful PCR amplifications from 113 *C*. *trachomatis* positive clinical specimens.

*Ct*GEM typing		Sequencing		Number (%)
rg1	ofr	rg1	ofr	
+	+	+	+	42 (37.2)
+	+	-	-	10 (8.8)
-	+	+	+	5 (4.4)
-	-	+	+	1 (0.9)
-	+	-	-	4 (3.5)
+	-	-	-	10 (8.8)
-	-	-	-	41 (36.3)

**Totals**				
*Ct*GEM typing				52 (46.0)
Sequencing				48 (42.5)
*Ct*GEM rg1				62 (54.9)
*Ct*GEM ofr				61 (54.0)

## Discussion

The accumulation of complete genome information from large numbers of isolates within a bacterial species makes it possible to design genotyping methods with precisely designed performance. However, this raises the question as to whether the recent very large cost reductions in whole genome sequence analysis have largely rendered bacterial genotyping methods obsolete. Our position is that there is a role for genotyping methods for high throughput surveillance or research, provided they are considerably faster and cheaper than genome sequencing, and yield useful information. *Ct*GEM typing encompasses two PCR reactions for each of two loci, using a generic SYBR green master mix. The procedure can be completed in three hours, and has a consumables cost of <AUD$3.00 per assay, not including any costs associated with nucleic acid purification. This is much faster and cheaper than whole-genome based methods. Furthermore, *C*. *trachomatis* is difficult and time consuming to culture, which makes methods based on direct analysis of clinical specimens especially useful. Direct genome sequencing of *C*. *trachomatis* from clinical specimens remains a significant technical task [3], so expanding the potential role for non-sequencing based methods. Most other *C*. *trachomatis* genotyping typing methods either target *ompA* alone and are frequently complex [30–32], or are MLST or variable number tandem repeat based methods [33–38] that in terms of cost and time taken may now have difficulty in competing with full genome-based methods. Very low cost and rapid methods for *C*. *trachomatis* genotyping are in general designed only to identify specific subgroups [39, 40]. For example the method developed by Schlaeffer and Henrich [40] resembles *Ct*GEM typing in that it targets *ompA* and one other gene, but it provides typing information only within the LGV lineage. *Ct*GEM provides a unique synthesis of low cost, simplicity, and species-wide informative power. *Ct*GEM typing is not suitable for contact tracing, or the inference of high resolution transmission networks, but would be highly effective for low cost monitoring of major lineages. We suggest that the availability of *Ct*GEM typing and whole genome sequence analysis could underpin most conceivable *C*. *trachomatis* surveillance tasks.

The key driver for the development of *Ct*GEM typing was the facilitation of the high throughput screening of *C*. *trachomatis* positive UGT specimens for the presence of known ocular strains [15, 16]. The two fragments selected for inclusion in the method make it particularly effective for this purpose. We took a conservative approach of defining only eight HRMA alleles of *ompA* meaning that not all omA genotypes are discriminated by the ofr HRMA. However, in a fortuitous manner, the rg1 HRMA not only discriminates the ocular from the UGT strains in these groups, but also discriminates the Australian ocular strains from the classical ocular strains. The effectiveness of the method is enhanced by the absolute correlation between the rg1 GC allele and the classical ocular and LGV lineages, and the rarity of *ompA* genotype D strains that have the rg1 GT allele. Both *ompA* genotype D and the rg1 GT allele are common in the *C*. *trachomatis* T1 lineage, but they very rarely coincide to generate *Ct*GEM type 9 false positives for the ocular AusBa strain.

Not only does *Ct*GEM typing identify specific ocular strains, it can also assign strains to the T1, T2, classical ocular, and LGV lineages. The rg1 GA allele is a virtually 100% sensitive marker for the T2 lineage. The specificity is less because there is a lineage of *ompA* genotype E strains in the T1 lineage that also has the rg1 GA allele. However, there are no known genotype E strains in the T2 lineage, so the combination of the GA allele in rg1 and “not E” at *ompA* is very sensitive and specific for the T2 lineage. Conversely, a GT or AT allele at rg1, or a combination of the *ompA* genotype E allele and the rg1 GA allele is very sensitive and specific for the T1 lineage. The rg1 GC allele is sensitive and specific for the classical ocular and LGV lineages. Within the strains that possess the rg1 GC allele, the *ompA* HRMA method generates unique curves for A, Ba, B, C, and L1/2 (LGV) *ompA* genotypes.

It is inevitable that such a streamlined method as *Ct*GEM typing has limitations. First, given that the method is based on known genetic variation, it is inherently vulnerable to being compromised by undiscovered variation. However, the recently publication of hundreds of *C*. *trachomatis* genome sequences from all over the world provided an extremely valuable resource [3]. We demonstrated the relationship between *Ct*GEM typing and population structure defined by the smaller set of genomes used to design the method remains valid with the larger set of genomes. Also, while there is concordance between *Ct*GEM type relative abundances in the global set of genomes, and clinical specimens from Queensland, the global set of genomes contained no strains of *ompA* genotype Ja, whereas these are quite common in the Queensland set. Further, one Queensland *ompA* genotype Ja strain possessed a rg1 GT haplotype (as opposed to the expected T2 associated GA haplotype that was found in the other Queensland *ompA* genotype Ja strains). The combination of rg1 haplotype GT and the ofr CHIaJJaK allele is *Ct*GEM type 11, the same as the AusC strain. Therefore this strain constitutes a single false positive for the ocular AusC isolates, that is absent from the global set of genomes [3]. As discussed below, ofr sequencing would show that this strain is not AusC.

The global set of genomes defined only a small number of additional SNPs in the regions subject to HRMA, and these were in very rare strains, The predicted effects on HRMA are to produce novel curves rather than to result in HRMA allele mis-calling. It may be that in the future the CtGEM allele designations and genotype numbering system will be expanded. However, the current protocol is predicted to be valid for the vast majority of extant *C*. *trachomatis* strains.

The *Ct*GEM data analysis protocol specifies that *ompA* genotypes C, H, Ia, J, Ja, and K are not discriminated from each other. We regard this as acceptable because these *ompA* variants are closely related [41–43] and the UGT associated *ompA* genotypes in this group (i.e. not C) are associated with the T2 lineage and appear to have similar distributions and ecology [1, 3]. Sequencing ofr with the CHIJJaK s HRMA curve would resolve the fragment into four classes composed of *ompA* genotypes C, K, Ja, and H+Ia+J (Fig 2). *OmpA* genotypes H, Ia, and J have identical ofr sequences so cannot be discriminated by sequencing. Importantly for screening for ocular strains based on current knowledge a T at position 67 in the ofr is 100% sensitive and specific for genotype C. We have observed that the ofr’s from genotypes C, K, and Ja melt at a lower temperature than those from genotypes H, and J (and inferred from Ia which has an identical ofr sequence). However, we have taken a conservative approach and not incorporated this into our protocol for data analysis, primarily because this still fails to provide 100% specificity for *ompA* genotype C. We therefore suggest sequence analysis of the ofr if more discrimination is needed, in particular when confirming the identification of an *ompA* genotype C strain strain.

A critical aid to the interpretation of the ofr HRMA is a conspicuous early melting domain in the ofr’s from genotypes C, H, Ia, J, Ja and K (experimentally demonstrated for all except *ompA* genotype Ia, which has an identical ofr sequence to genotypes H and J). The ofr from *ompA* genotype A has an identical sequence to *ompA* genotypes H, Ia, and J except for a T→C substitution at ofr position 140, and although there is evidence for an early melting domain from *ompA* genotype A, it is much less conspicuous a than in HRMA curves from C, H, Ia, J, Ja, and K. This suggests that the existence of the conspicuous early melting domain is largely dependent on a T at position 140. The association between the allelic state of the position 140 T-C SNP and the nature of the early melting domain is only seen with the closely related *ompA* genotypes A, C, H, Ia, J, Ja, and K derived ofr sequences. Apart from the ofr derived from *ompA* genotype L3, all other known *ofr* variants possess a T at position 140, and yet do not have the ofr early melting domain. This is explicable as there are many differences between ofr sequences from *ompA* H, Ia, J, Ja, K, and the other variants of *ompA*, which could easily change the melting domain structure. Further, there was complete concordance between our observations of early melt domain magnitude and presence/absence, and *in silico* prediction using the software uMELT [44] (data not shown).

A minor limitation of the study is that we have not fully defined the ability of *Ct*GEM typing to identify *C*. *trachomatis* LGV strains. We experimentally established that the *ompA* L2 genotype ofr generates a unique HRMA curve, and the ofr’s from *ompA* genotypes L1, L2 and L2b are identical, so these *ompA* genotypes should all be identified. An exception is the very rare aberrant *ompA* sequence from strain L1-440, which is predicted to be indistinguishable from classical ocular genotype Ba by *Ct*GEM typing. We have not tested any L3 strains, which have an ofr very similar to *ompA* genotype A, differing only in having a C instead of a T at ofr position 127. We predict, using both uMELT [44], and comparison with observed curves, that *ompA* genotype L3 will yield an ofr melting curve similar but not identical to genotypes A, E and G. However, LGV strains all possess the rg1 GC haplotype, whereas genotypes E and G strains with rg1 haplotype GC have not been found. Genotype A and L3 strains share the rg1 GC haplotype so are potentially difficult to discriminate by *Ct*GEM typing. However genotype A strains are ocular, while LGV strains are associated with invasive STIs, and genotype L3 and A strains can be discriminated by ofr sequencing. Therefore, we feel this limitation is manageable.

There was a failure rate of 54% when the HRMA method was tested against clinical specimens, with failure being almost entirely due to lack of PCR amplification. The proportion of failures, and the identities of the specimens that failed were very similar for *Ct*GEM method, and for amplification followed by sequencing of the same loci. This suggests that PCR amplification failure is a consequence of low *C*. *trachomatis* DNA concentration. Amplification sensitivity is a common issue with *C*. *trachomatis* typing methods based on direct PCR amplification from clinical specimens, probably because of great variability in the mass of *C*. *trachomati*s DNA in clinical specimens, and the high sensitivity of commercial diagnostic systems which often target the multicopy resident plasmid and make use of larger volumes of purified nucleic acid than the 1μl used by us. It was noted that failure in a second stage PCR was always preceded by undetectable amplification in first round PCR, whereas with successful amplification, amplimer was always detectable after the first round PCR. Our reasoning is amplification failure is due to complete failure of amplification to commence, and the primary function of adopting a nested or semi-nested PCR formats is not to maximise amplification sensitivity, but to achieve consistency in final PCR yield and so enhance the HRMA robustness. There is scope for improvement of PCR sensitivity. First, it was not uncommon to see amplification failure of one or other of the loci, suggesting that the PCR reactions for one locus are not inherently more sensitive than for the other. Rather it indicates there is a stochastic element to failure that could be addressed by repetition of the amplification reactions. Second, concentrating the nucleic acid purified from the specimens, and/or increasing the nucleic acid solution volume in the reaction, may increase sensitivity.

In conclusion, we have developed and demonstrated HRMA-based *Ct*GEM typing, a low cost and efficient means of subdividing *C*. *trachomatis* into types concordant with phylogeny and anatomical tropism. *Ct*GEM typing is likely applicable to *C*. *trachomatis* surveillance in research or public health contexts. If used for screening for ocular strains, it has 100% sensitivity and sufficient specificity to exclude the great majority of ocular strains. Further identification of ocular strains can be confirmed by sequencing of ofr.

## Materials and methods

### *C*. *trachomatis* isolates and specimens, and DNA extraction

*C*. *trachomatis* isolates (Aus3, Aus9, Aus11, Aus15, Aus19, Aus20, and Aus25) were obtained from a study of mother and child pairs conducted in the Northern Territory, Australia between 1985 and 1993 [2, 17]. DNA was extracted from three 25cm^3^ culture flasks at 80–100% infectivity stored at -80°C using a QIAamp DNA Mini Kit (Qiagen, Chadstone, Australia).

Genome-sequenced *C*. *trachomatis* reference strains A2497 [1] and UCH1 [45] were obtained from the *Chlamydia* Biobank, University of Southampton, UK. The strains were cultured in HeLa cell monolayers which were propagated in DMEM supplemented with 10% foetal calf serum. The inocula were centrifuged onto a confluent monolayer at 750xg for 30 minutes at room temperature. Infected cultures were grown in DMEM with 10% foetal calf serum plus 1μg/mL cyclohexamide and 20μg/mL gentamicin to 80–100% infectivity. Elementary bodies were harvested from three 25cm^3^ culture flasks by scraping the cells from the surface of the flask and centrifuging at 3000xg. The cell pellet was resuspended in cold PBS containing glass beads and vortexed at high speed for 1 minute to lyse the cells. Cell debris was removed by centrifugation at 250xg for 5 minutes and DNA was extracted from the elementary bodies in the supernatant using a GenElute Bacterial Genomic DNA Kit (Sigma-Aldrich, Sydney, New South Wales).

PS297 is a *C*. *trachomatis*-positive diagnostic urogenital swab specimen, collected as part of a prospective study of *C*. *trachomatis* genotypes in the Northern Territory, Australia [16]. Royal Darwin Hospital diagnostic laboratory staff extracted DNA from the specimen using the Siemens Versant system (Siemens Healthcare Australia, Bayswater, Victoria).

DNA from reference strain ATCC VR-902B was purchased as Amplirun® *C*. *trachomatis* DNA control (Vircell, Madrid, Spain).

*C*. *trachomatis*-positive clinical samples from Queensland, Australia were collected as part of a study to understand current serovar and genetic differences in ocular and urine samples. Specimens were urine remnant DNA extracts from the Roche 4800 CT/NG test (Roche Diagnostics, Australia).

### Identification of informative SNP combinations

We used a previously described genome-wide orthologous SNPs matrix inferred from whole genome sequencing data from 65 diverse *C*. *trachomatis* isolates [2]. The orthologous SNP matrix was used as input into the computer program “Minimum SNPs” [26, 27]. This program can derive sets of SNPs from DNA sequence alignments based on maximised Simpson’s index of Diversity (D). In this context, D is the probability that two known sequence variants chosen at random from the alignment (without replacement), will be discriminated by the SNP set under test. Combinations of SNPs were assessed for informative power *in silico*, to determine a maximally efficient means of meeting the assay specifications. This process included manual investigations of the feasibility of PCR primer design, and the potential effects of other nearby SNPs which have the potential to add resolving power, or conversely, confound an HRMA assay if the SNPs are linked and have opposing effects on amplimer G+C content.

### Genotyping procedure

The optimised method encompasses the HRMA analysis of two DNA fragments, one within *ompA*, and the other within a universally present putative membrane protein-encoding gene that is annotated as Jali_1891 in the genome of isolate B_Jali20, which we designated “region 1”. The format was a nested (region 1) or semi-nested (*ompA*) PCR in which the products of the first round PCR were subjected to a second round of PCR, followed by HRMA. For the region 1 fragment, an unlabelled probe and second round asymmetric PCR were used to facilitate discrimination of known variants.

The primer and probe sequences, and PCR and HRMA temperature conditions are shown in Table 4. All PCR reactions were performed in a QIAGEN Rotorgene device using Platinum® SYBR® Green qPCR SuperMix-UDG chemistry (Invitrogen) with the addition of 5% Q-solution (5M betaine) in a total volume of 10μl. For the *ompA* fragment (ofr), the first round PCR contained 0.4μM of each primer and 1μl of DNA extraction. The second round PCR and HRMA reaction contained 1μl of the first round PCR product and 0.4μM of each primer. For the region 1 fragment (Rg1), the first round PCR contained 0.5μM of each primer and 1μl of DNA extraction. The second round PCR and HRMA reaction contained 1μl of the first round PCR product, 0.04μM of the forward primer, 0.4μM of the reverse primer, and 1μM of the probe. A detailed account of the assay design and method development is given in S1 File. Isolates of known genotype that were used in the development of the assay are shown in Table 5. The work-flow for analysing the HRMA data is provided in S2 File.

**Table 4:** PCR primer and probe sequences, and PCR and HRMA conditions.

Region name	PCR reaction	Oligo	Oligo sequence (5’-3’)	Product size (bp)	PCR and HRMA conditions	HRMA normalisation regions (T^o^C)
rg1	First round	FR	CATCCACTTTACCAGCGATTC	193	50°C 2min; 95°C 2min;50 cycles of 95°C 10secs, 61–56°C (1°C per cycle) 10secs, 72°C 20secs;95°C 1min; 40°C 1min	
			CCATCTTTGGCTCGTGCTAT			
	Second round(nested)	FRprobe	GGAAAGGTCAGCACTCTTGAA	74	50°C 2min; 95°C 2min;50 cycles of 95°C 10secs, 61–56°C (1°C per cycle) 10secs, 72°C 20secs;95°C 1min; 40°C 1min;50–95°C in 0.1°C increments for 2secs	53–54°C80-81°C
			GGGGTTCTTGTTGTTTTAGGG			
			TAGCTAGATAGGCTATCCGAAAACAAATCC[Phos]			
	Sequencing	FR	CCCATTGCCGAGAGATAAAA	354		
			CTCCTGCGGAGGTTAGATTG			
ofr	First round	FR	TCCTACTGCAATACCGCAAG GCTTCTTTCCAATATGCTCAATC	459	50°C 2min; 95°C 2min;50 cycles of 95°C 10secs, 59–54°C (1°C per cycle) 10secs, 72°C 40secs;95°C 1min; 40°C 1min	
	Second round(semi-nested)	FR	GCCATTCATGGTAATCAATAGAG GCTTCTTTCCAATATGCTCAATC	184	50°C 2min; 95°C 2min;50 cycles of 95°C 10secs, 59–54°C (1°C per cycle) 10secs, 72°C 40secs;95°C 1min; 40°C 1min;65–95°C in 0.1°C increments for 2secs	73–74°C83-84°C
	Sequencing	FR	TCCTACTGCAATACCGCAAG	1015		
			TGAACCAAGCCTTATGATCG			

**Table 5:** Control isolates used during development of *Ct*GEM typing.

**ofr controls**		**Genome accession**	**ofr genotype**	**ofr curve**
	A2497	FM872306	A	A
	Aus3	ERS153020	B	BF
	Aus25	ERS351392	Ba	BaD
	Aus9	ERS153045	C	CHIJK
	Aus11	ERS153024	D	BaD
	Aus15^*a*^	-	E	E
	Aus20	ERS153031	F	BF
	Aus19	ERS153037	G	G
	UCH1	ERS001407	L2b	L
	**rg1 controls**	**Genome accession**	**rg1 genotype**	**rg1 curve**
	ATCC VR-902B	-	GC	GC
	Aus3	ERS153020	GA	GA
	Aus9	ERS153045	GT	GT
	PS297^*b*^	-	AT	AT

^*a*^ Aus15 was serotyped as a serovar E [2, 17] and shown to have an identical ofr curve to clinical samples analysed in this study which had an ofr E sequence.

^*b*^ PS297 is a clinical sample [16] for which rg1 was sequenced and confirmed as an AT genotype.

### Sequencing of rg1 and ofr from clinical specimens

Each region was initially amplified by PCR using the primers described in Table 4. PCR products were purified and Sanger sequenced by service providers, the Australian Genome Research Facility (Brisbane, Australia).

### *In-silico* assessment of assay performance

The original 65 genome dataset [2] and additional genome sequence data from a global study of *C*. *trachomatis* phylogeny [3] were analysed *in silico* to determine their *Ct*GEM type. A reference set of known genotypes from the 65 genomes [2] used in the design of the assay, was generated for each of the two HRM fragments. For ofr, the reference genotypes were 184bp which corresponded to the *ompA* fragment HRM amplicon. For rg1, the reference genotypes were 400bp which encompassed the 74bp of the region 1 HRM amplicon. Short read genome sequence data for individual isolates was aligned against the reference genotypes for ofr and rg1 using ARIBA [29]. Of the 563 genomes in the global study [2], 63 were already included in our original dataset of 65 genomes, and 2 isolates we were unable to obtain short read data for (D_UK750364 and E_UK220880), providing an additional 498 genomes for analysis. Of the 563 genomes thus analysed in total, 4 did not produce a result for one of both or the *Ct*GEM fragments (B_Aus6, F_Swab5, L2_P, and A_MH20130), yielding complete *Ct*GEM predictions for 559 isolates.

## Ethics

This study was approved by the Human Research Ethics Committee of the Northern Territory Department of Health and Menzies School of Health Research (permit number 10–1337) and the Human Research Ethics Committee of the University of Queensland (permit number 2015000301).

## Supporting information

S1 File Method development. Additional information regarding development of the *Ct*GEM method.
(DOCX)

S2 File HRMA curve interpretation. A detailed protocol for HRMA curve interpretation.
(DOCX)

S3 File Rg1 sequences. Rg1 sequences derived from the Queensland specimens.
(TXT)

S4 File Partial *ompA* sequences. Partial *ompA* sequences derived from the Queensland specimens.
(TXT)

S5 File Complete HRMA results. Genotypes inferred from HRMA, and HRMA raw data.
(XLSX)
