# Supplementary material for: CtGEM typing: Discrimination of Chlamydia trachomatis ocular and urogenital strains and major evolutionary lineages by high resolution melting analysis of two amplified DNA fragments
Source: PLoS One. 2018 Apr 10;13(4):e0195454. doi: 10.1371/journal.pone.0195454 (PMC5892870; doi:10.1371/journal.pone.0195454)
Supplement: S1 File — Additional information regarding development of the CtGEM method. (DOCX) [file pone.0195454.s001.docx]

**Detailed account of assay design and method development.**

The resource used to identify useful combinations of SNPs was the genome wide orthologous SNP matrix, referred to in the Methods. Modifications to the SNP matrix were made to circumvent format constraints on Minimum SNPs input files: all positions for which there were gaps in any of the rows were removed, and all positions which were classified as “N” on the basis of sequence quality score failure were converted to “G”. The chromosomal positions of SNPs candidates for inclusion in a genotyping method were identified in the “custom reference genome” referred to in the Methods. A whole genome alignment of all 65 genomes was reconstructed from the custom reference and the SNP matrix. This alignment was used to assess the feasibility of designing HRMA assays incorporating SNPs of interest. SNPs selected for further progression in the assay development were then converted to chromosome positions in the closed annotated *C. trachomatis* B/Jali20 genome sequence (NC012686).

We explored the genome-wide orthologous SNP matrix (described in Methods) using the Minimum SNPs software, to identify SNP sets with high discriminatory power. This revealed that the SNP defined by position 222834 in the in the B_Jali20 genome appeared particularly useful. This SNP is tri-allelic and is within a hypothetical gene (Jali-1891) that is annotated as likely encoding a membrane protein. Our initial observation from Minimum SNPs analyses was that SNP22834 in combination with any of a number of SNPs in *ompA* provided the best possible predicted resolving power of a two SNP-based genotyping assay. We also noted that the alleles of SNP22834 correspond to deep phylogenetic lineages. Because the required performance of our genotyping method was to resolve deep phylogenetic lineages, and also resolve lineages that may be closely related with respect to genome-wide orthologous SNPs but have acquired disparate *ompA* genes, we decided to design a genotyping method based on SNP22834 and *ompA*.

Examination of aligned Jali_1891 sequences revealed an additional SNP at position 222832 (using B_Jali20 genome numbering) i.e. two positions away from the SNP identified using Minimum SNPs. The distribution of the alleles of these SNPs defines four haplotypes: GA, GT, GC, and AT, (with the first SNP being position 22832 and the second position 22834). Further examination indicated that these haplotypes are superior to SNP 22834 on its own in indicating the major evolutionary lineages, and that it was feasible to design PCR primers to amplify a region contain both SNPs with a view to HRMA. Next we examined *ompA*. Although a number of highly discriminatory SNPs were identified within *ompA* using Minimum SNPs, none of these were within regions that were feasible for HRMA, with nearby SNPs either making PCR primer design impossible or predicted to confound HRMA interpretation. As an alternative approach we hypothesised that a fragment previously reported to discriminate *ompA* genotypes B and Ba by HRMA (1), may be more widely applicable in discriminating *ompA* variants. Examination of known sequence variation in this fragment revealed numerous SNPs that defined %G+C classes approximately in accordance with *ompA* genotypes, and also that the previously identified PCR primers would be predicted to be functional in all known *C. trachomatis*. We therefore concluded that a double locus HRMA assay based on this previously described *ompA* fragment, and the four haplotypes defined by the SNPs at B-Jali20 positions 22832 and 22834 may would provide the required performance of discriminating major evolutionary lineages and identifying ocular *ompA* variants.

To implement this method, it was first necessary to design an HRMA assay that resolved all four known haplotypes of region 1. The haplotypes define only three %G+C classes, with the GA and GT haplotypes predicted to be difficult to resolve by HRMA of the entire amplimer. Accordingly we designed an unlabelled probe predicted to form a duplex with one of amplimer strands. This duplex would melt at a lower temperature than the entire amplimer and provide haplotype information. The probe is 100% identical to the GA haplotype (so hybridizes to the complementary strand), and has a single mis-match with the GT haplotype, so should allow discrimination of the GA and GT haplotypes on the basis of probe duplex Tm. The GT and GC haplotypes both have one mismatch with the probe, but we predicted that these would be resolved on the basis of Tm of the entire amplimer. Similarly, the AT haplotype would be predicted to be resolved from the other haplotypes on the basis of two mismatches with the probe, and also the lowest amplimer %G+C. This strategy necessitates the production of single stranded amplimer in the later stages of the PCR (asymmetric PCR) which is accomplished by using unequal primer concentrations (see methods).

Initially laboratory experiments were carried out using a set of cultured *C. trachomatis* and also clinical specimens that had been subjected to *ompA* genotyping by a Taqman hydrolysable probe-based method (refs). We first attempted optimisation the Region 1 reaction, using a non-nested format with the inner primer set only (see Methods). The robustness of this format was unacceptably poor, primarily because of inconsistency as to whether probe melting could be discerned in the HRMA curve. This was despite extensive efforts to optimise PCR primer concentration. A correlation between poor PCR amplification and inability to discern probe melting was observed (data not shown), and it was reasoned that when PCR yield is low, there is insufficient primer depletion for the PCR reaction to become asymmetric and yield single stranded DNA that would bind the probe. Accordingly, the method was redesigned in a nested format to increase the probability that final PCR yields are high. This strategy was successful, and after some optimisation of the second stage reaction primer concentrations, the procedure described in the Methods was arrived at. The predicted variation in the probe duplex and amplimer Tm values were observed (Fig. 1).

Next we tested the *ompA* reaction using a similar set of specimens. Once again initial experiments were with a non-nested format using only the inner primer set. After adopting a nested format for the region 1 reaction, we also developed a nested format for the *ompA* reaction. Although the *ompA* reaction does not involve a probe, the nested reaction provided superior Tm reproducibility (data not shown).
