# Supplementary figures and images for: CtGEM typing: Discrimination of Chlamydia trachomatis ocular and urogenital strains and major evolutionary lineages by high resolution melting analysis of two amplified DNA fragments
Source: PLoS One. 2018 Apr 10;13(4):e0195454. doi: 10.1371/journal.pone.0195454 (PMC5892870; doi:10.1371/journal.pone.0195454)

**Data S2 Detailed method for analysis of ofr curves.**

STEP 1


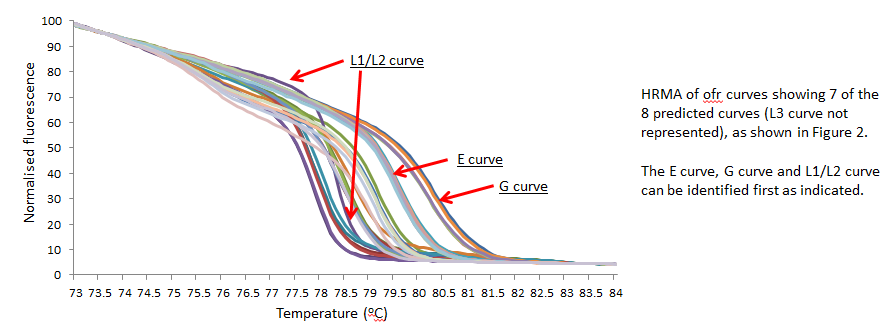


STEP 2


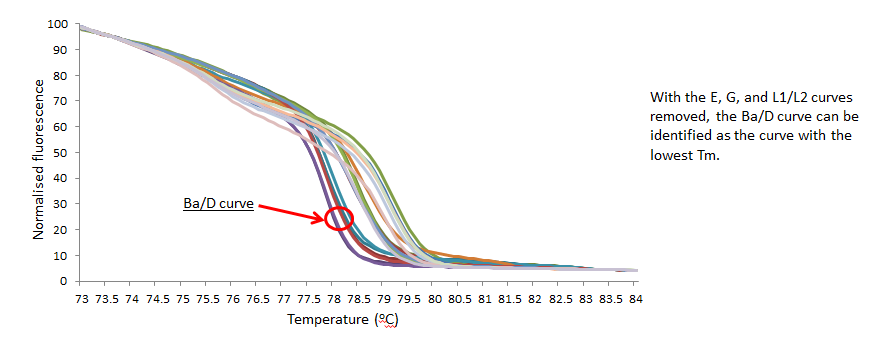


STEP 3


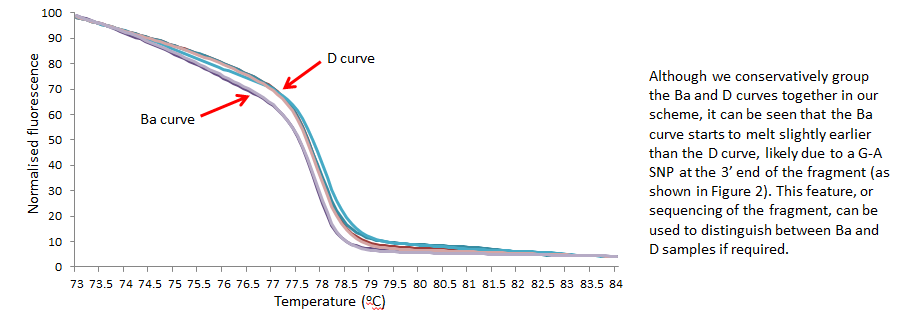


STEP 4


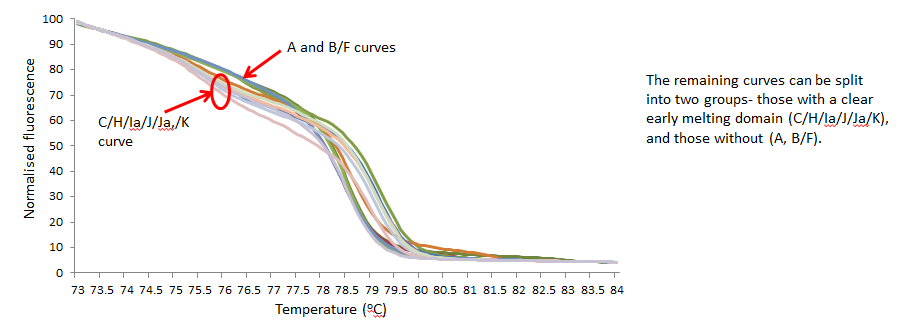


STEP 5


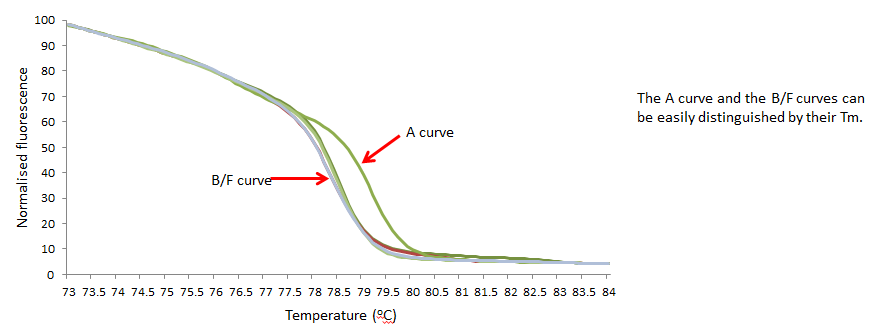


STEP 6


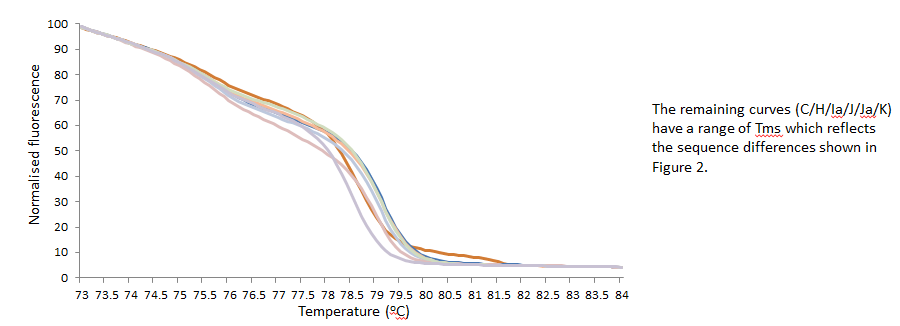

Supplement: S2 File — A detailed protocol for HRMA curve interpretation. (DOCX) [file pone.0195454.s002.docx]
